# A guide to the reporting of protocols of pilot and feasibility trials

**DOI:** 10.1186/s40814-019-0423-8

**Published:** 2019-02-28

**Authors:** Lehana Thabane, Gillian Lancaster

**Affiliations:** 1 Department of Health Research Methods, Evidence, and Impact, McMaster University, Hamilton, ON Canada; 2 Institute of Primary Care and Health Sciences, Keele University, Keele, UK

## Abstract

Publishing protocols of trials including protocols of pilot and feasibility trials—designed to inform the designs of main trials—has been advocated as an important strategy towards improving transparency in the conduct and reporting of main trials and pilot/feasibility trials. This editorial aims to provide some general guidance on how to report protocols of pilot and feasibility trials, drawing upon two available resources—the CONSORT extension to pilot trials and the SPIRIT guideline for main trials. We describe how these might be adapted for the reporting of protocol manuscripts of pilot and feasibility trials for submission in *Pilot and Feasibility Studies* journal.

## Introduction

The journal *Pilot and Feasibility Studies* (*PFS*) was launched in 2015 to provide a forum for publishing pilot and feasibility research. PFS’s scope “encompasses all aspects of the design, conduct and reporting of pilot and feasibility studies in biomedicine. The journal publishes research articles that are intended to directly influence future clinical trials or large-scale observational studies, as well as protocols….” [1]. First published in 1996 [2] and updated in 2010 [3], the CONSORT Statement was developed to improve the transparency of the reporting of randomised controlled trials (RCTs). CONSORT was then extended to cover various types of study outcomes [4, 5], RCT designs [6–9], and types of interventions [10–12]. In 2016, we developed the CONSORT extension to pilot trials to help researchers who conduct pilot and feasibility trials to report them in a way that allows readers to understand what had been done, what the results were, and how to interpret them [13–15]. For the results of pilot and feasibility trials, PFS now requires that submissions include the checklist from the CONSORT extension to pilot trials [1].

Publishing protocols of trials has been advocated as an important strategy towards improving transparency in the conduct and reporting of trials [16, 17]. This has allowed investigators to assess consistency between protocols and final reports of trials [18], as a way to hold authors more accountable and to help readers to better assess the validity of the final trial results. Most importantly, it has helped readers to better understand the conduct and applicability of the trial and better apply the results to patient care.

Today, publication of trial protocols in scientific journals has become more commonplace. The SPIRIT (Standard Protocol Items: Recommendations for Interventional Trials) guideline was published in 2013. It aims to improve the completeness and quality of reporting clinical trial protocols [19]. SPIRIT defines a protocol as “a document that provides sufficient detail to enable understanding of the background, rationale, objectives, study population, interventions, methods, statistical analyses, ethical considerations, dissemination plans, and administration of the trial; replication of key aspects of trial methods and conduct; and appraisal of the trial’s scientific and ethical rigour from ethics approval to dissemination of results” [19]. The guideline includes a full statement, a checklist of items that should be included in a trial protocol publication, and a detailed explanation of the importance of each item, with examples of best practices [20].

Reporting guidelines such as SPIRIT have several potential benefits to various stakeholders [21]: (1) for funding agencies—to use guideline items as part of a grant submission template and evaluation criteria (e.g. Swiss National Science Foundation [http://www.snf.ch/SiteCollectionDocuments/IICT_2018_Proposal_Template.docx, http://www.snf.ch/en/funding/programmes/iict/Pages/default.aspx#Documents] and National Health and Medical Research Council [https://www.australianclinicaltrials.gov.au/clinical-trials-toolkit]); (ii) for authors—to use the guideline checklist as a template when preparing trial manuscripts for publication; (iii) for peer-reviewers of journal manuscripts—to endorse the guideline in their instructions to authors and to require the guideline checklist to be included as part of their submission process (e.g. Trials [https://trialsjournal.biomedcentral.com/]) and BMJ Open [https://bmjopen.bmj.com/]); and (iv) for higher education institutions—to endorse guidelines and ensure that they are incorporated into their institutional research publication policies (see http://www.spirit-statement.org/about-spirit/spirit-endorsement/). The absence of reporting guidelines can be a serious barrier to the full and transparent dissemination of knowledge through scientific research—reflecting poorly on all stakeholders and potentially leading to biased reporting, waste of resources, and unusable research that is hard or impossible to replicate.

Against this backdrop, a call for publication of protocol guidance for pilot and feasibility studies was made in 2016 [22]. While this work has been planned by the same working group that developed the CONSORT extension to pilot trials, which includes ourselves, we recognise that some guidance from the journal editors would be helpful. This editorial aims to provide some general guidance in the interim, drawing upon two sources—the CONSORT extension to pilot trials and the SPIRIT guideline for main trials. We describe how these might be adapted for the reporting of protocols of pilot and feasibility trials.

## Guidance for writing protocols of pilot and feasibility trials for submission to PFS journal

Since its inception in 2015, the PFS journal has routinely published articles reporting protocols for pilot and feasibility studies. PFS’s policy is that protocol manuscripts should be submitted to the journal before the recruitment and follow-up of study participants have been completed. To date, there is no specific evidence-based guidance describing the items that should be included when reporting the protocol for a pilot and feasibility trial. We suggest that it is helpful to refer to the CONSORT extension to pilot trials for more elaboration on items to report at the start of the work when planning the study protocol, while concurrently adapting the SPIRIT checklist for reporting the protocol of a pilot trial.

However, SPIRIT is designed to comprehensively report main trials of effectiveness. This is not the primary aim of a pilot trial, which aims to test a set of feasibility objectives to ensure the main trial is viable. We suggest adapting SPIRIT for manuscripts that report the protocols of pilot and feasibility trials. The SPIRIT checklist can be found on the SPIRIT Statement website at http://www.spirit-statement.org/. For example, all Background elements of the SPIRIT guideline would be suitable to consider in the Background section of the protocol for a pilot trial, supplemented by the item from the CONSORT extension (item 2a) that specifies the reason for the pilot trial set in the context of the main trial. The CONSORT extension to pilot trials (item 2b) also stresses the importance of giving clear aims and feasibility objectives for the pilot trial at the end of the introduction. These would provide the main focus of the pilot trial protocol (replacing SPIRIT item 7).

In the Methods section, rather than describing the primary and secondary outcome measures as stipulated in SPIRIT (item 12), we should instead describe the methods for the primary and secondary feasibility outcomes, for example recruitment rate, compliance in data collection or acceptability of the intervention to patients and health practitioners, or secondary patient-centred outcome measures to be used for data collection (CONSORT extension item 6a). Similar adaptations should be made for SPIRIT item 14 (CONSORT item 7a) on sample size and SPIRIT item 20a (CONSORT item 12a) on data analysis. Thus, one can consider the SPIRIT elements in the context of assessment of feasibility as the primary aim for a pilot trial and therefore adapt them appropriately, supplemented with reference to the CONSORT extension explanations. It is important to note that all elements of the Background and Methods are generally part of the design of a trial, and therefore, they would typically be addressed in the protocol of the pilot trial, which is addressing any uncertainties in study design.

Headway has been made into producing a guideline for reporting pilot trial protocols. The next step of the working group is to convene a consensus meeting to discuss the suggested adaptations with the aim of producing a final checklist that can be used to write the statement and explanatory paper for the SPIRIT extension to protocols of pilot and feasibility studies. In the meantime, we hope the suggested adaptations will be useful to authors in reporting protocols of pilot and feasibility studies submitted to PFS.
